# Normal Anal Sensibility in Patients Born With Anorectal Malformations

**DOI:** 10.1111/nmo.14983

**Published:** 2024-12-31

**Authors:** Venla E. C. den Hollander, Monika Trzpis, Paul M. A. Broens

**Affiliations:** ^1^ Department of Surgery, Anorectal Physiology Laboratory University of Groningen, University Medical Center Groningen Groningen the Netherlands; ^2^ Department of Geriatric Medicine University of Groningen, University Medical Center Groningen Groningen the Netherlands; ^3^ Department of Surgery, Division of Pediatric Surgery University of Groningen, University Medical Center Groningen Groningen the Netherlands

**Keywords:** anal canal, anorectal malformations, innervation, pudendal nerve

## Abstract

**Objectives:**

Anal sensibility is crucial for fecal continence. We aim to investigate anal sensibility in patients diagnosed with different types of ARM, after various types of treatments, and according to the sacral ratio.

**Methods:**

Retrospectively, we included 68 ARM patients (7 treated non‐surgically and 61 surgically). All underwent anorectal function tests. We measured anal sensibility, the existence of a functional internal anal sphincter (IAS), and sacral ratio.

**Results:**

The mean anal sensibility was 7.2 (SD 5.2). Normal anal sensibility was present in 40/60 patients, irrespective of the malformation type. Patients diagnosed with minor types had better anal sensibility than those with major types (5.1 vs. 8.4 mA, respectively, *p* = 0.007) and those treated non‐surgically vs. surgically (2.7 vs. 7.7 mA, respectively, *p* = < 0.001). Anal sensibility was significantly better in patients who possessed an IAS than in patients without an IAS (6.4 and 9.2 mA, *p* = 0.038). Multivariable regression analysis demonstrated a relationship between anal sensibility and the existence of a functional IAS. Anal sensibility was not associated with the sacral ratio.

**Conclusions:**

Normal anal sensibility can be present in ARM patients diagnosed with all types of ARM after they have been treated with corrective surgery. Anal sensibility was better in those with a functional IAS. This means that the IAS, present in the distal end of the fistula, should be spared as much as possible to preserve anal sensibility. In this way, aiming to maintain the best possible fecal continence.

Normal anal sensibility can be present in ARM patients diagnosed with all types of ARM after they have been treated with corrective surgery. Anal sensibility was better in those with a functional IAS. This means that the IAS, present in the distal end of the fistula, should be spared as much as possible to preserve anal sensibility. In this way, aiming to maintain the best possible fecal continence. Furthermore, the outcomes of this study demonstrate that anal sensibility is regulated by transmural nerves.

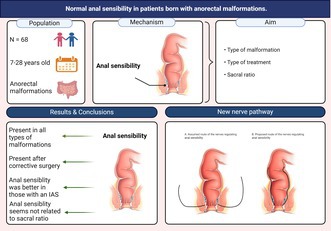

**Abbreviations**
ARM
: anorectal malformation
ASARP
: anterior sagittal anorectal plasty
IAS
: internal anal sphincter
PSARP
: posterior sagittal anorectal plasty
RAIR
: rectoanal inhibitory reflex

**Summary**

- Anal sensibility can be present in all types of anorectal malformations (ARMs) and irrespective of the sacral ratio
- Anal sensibility seems to be better in patients with ARMs that underwent corrective surgery during which the anal canal (including the internal anal sphincter) was transferred within the external anal sphincter complex
- As much as possible, the fistula should be transferred into the external anal sphincter during corrective surgery to maintain anal sensibility and, in this way, improve long‐term outcomes regarding fecal continence.
- Anorectal surgeries, where the rectum is dissected just above the anal canal, may have to be redesigned because this type of operation will denervate an essential part of the anal canal sensibility and therefore may result in fecal incontinence.

## 1 Introduction

Patients born with anorectal malformations (ARMs) can suffer from fecal incontinence (FI). A recent systematic review demonstrated that the prevalence of FI can reach up to 76% [1]. One of the causes of FI in these patients is decreased or absent anal sensibility [2]. Anal sensibility is essential to fecal continence by differentiating between different forms of fecal matter [3]. It is the ability to sense gas, liquid stool, and solid stool through anal receptors in the anal canal [4, 5, 6]. The ability to perceive and differentiate anal contents will allow the patient to anticipate defecation, activate the voluntary continence mechanisms to hold stool or relax the voluntary mechanisms to allow defecation. However, the ability to sense anal contents in the anal canal in patients born with ARM has only been investigated scarcely.

The bowel of patients born with ARM terminates outside the center of the external anal sphincter [7]. The majority of these patients need corrective surgery whereby the bowel is placed in the center of the external anal sphincter. During this procedure, the distal end of the bowel needs to be dissected extensively from the surrounding tissue [8, 9]. The distal end of the bowel in these patients is referred to as a fistula, and studies describing this part of the bowel state that it lacks the continence mechanisms present in healthy individuals [10, 11]. Therefore, this distal end is sometimes resected during corrective surgery, and the remaining bowel is placed in the center of the external sphincter. Nevertheless, recent studies demonstrated that irrespective of the malformation type, the distal end of the bowel contains a functional internal anal sphincter (IAS) [12, 13, 14]. This suggests that the distal end of the bowel is, in fact, an ectopic anal canal [13, 15, 16, 17]. Ruttenstock et al. also demonstrated, preoperatively, the presence of a functional IAS in the so‐called fistulous ending by measuring the rectoanal inhibitory reflex (RAIR). After resecting part of the fistulous ending; however, it appeared to be impaired [14]. If this distal end of the bowel is, in fact, an ectopic anus, it could mean that patients born with ARMs also have anal receptors that regulate anal sensibility in the anal canal. Some studies demonstrated that such patients can have proper anal sensibility after surgical treatment [2, 18]. They showed that patients with different types of ARM were able to sense electric stimuli during the anal mucosal electric sensations test. Those with minor types had a better anal sensory threshold (< 7 mA), while those with major types had worse anal sensibility (> 7 mA). Furthermore, they demonstrated that those with diminished anal sensibility were unable to differentiate gas from stool. Also, those diagnosed with FI had worse anal sensibility than those fecally continent. These studies included only a few patients, not all types of ARM, and not all types of surgical interventions were included. Therefore, there is a need for a more extensive study that provides for patients with different types of ARM and various kinds of treatments to investigate how anal sensibility is regulated in patients born with ARM.

Studies investigating the neurophysiological anatomy in healthy subjects describe that anal sensibility is regulated by the inferior rectal nerve. They believe that the anal canal is innervated by the inferior rectal nerve that carries the sensations from the anal canal to sacral levels 2, 3, and 4 [6, 19]. The inferior rectal nerve originates from the pudendal nerve, which courses bilaterally from the sacrum to the perineum and enters the perineum through Alcock's canal. In Alcock's canal, three nerves branch off the pudendal nerve; the inferior rectal nerve, the dorsal nerve that runs to the penis or clitoris, and the perineal nerve. The inferior rectal nerve then enters the anal canal distally and from the lateral direction through the anal canal wall. However, the neurophysiological mechanisms that regulate anal sensibility in patients born with ARM have never been described. Rectal sensibility has been thoroughly investigated in healthy subjects and patients born with ARM [12, 20, 21]. However, this article will only discuss anal sensibility.

**FIGURE 1:**
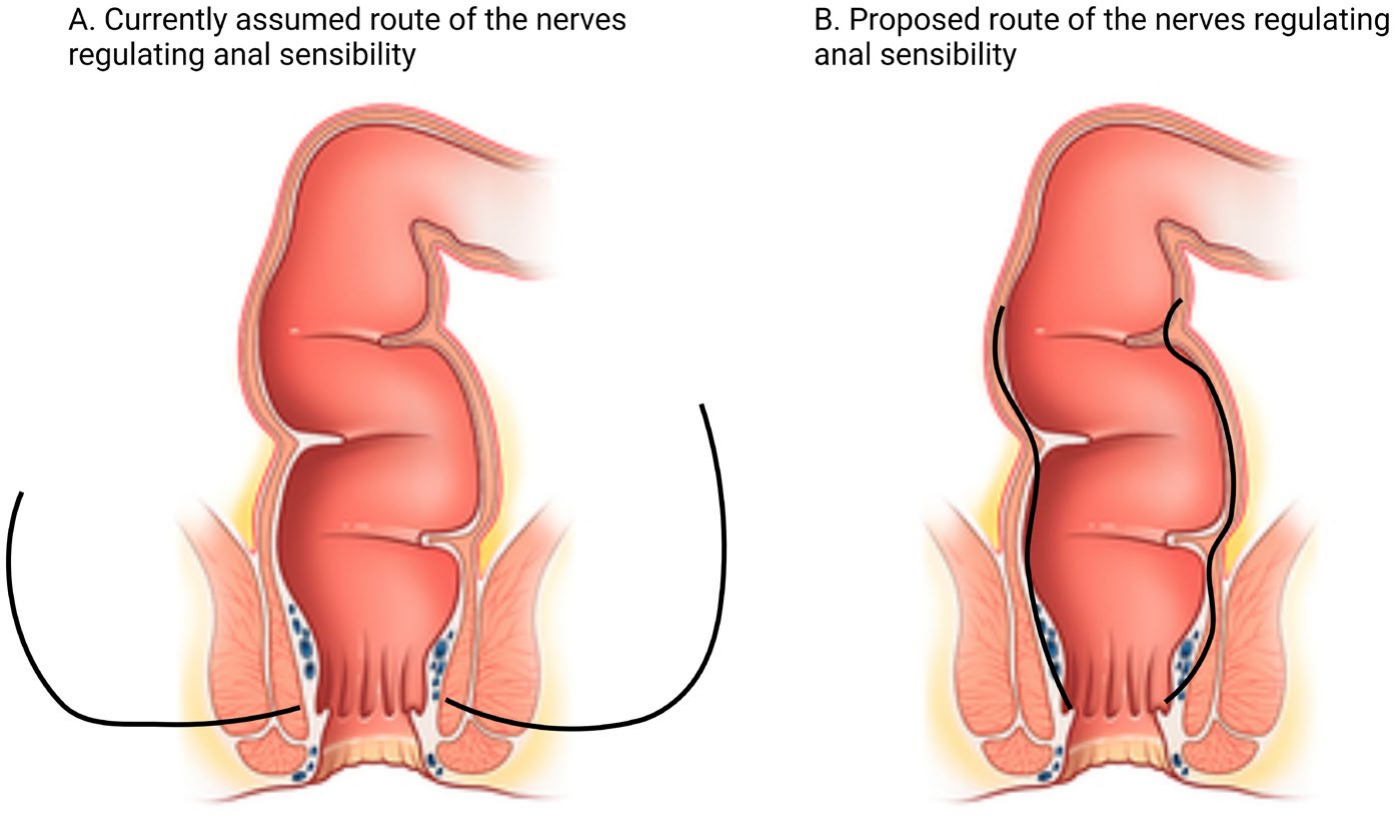
The innervation of the anal canal as currently found in the literature and as proposed by us. (A) Current literature states that the inferior rectal nerve regulates anal sensibility. This nerve originates from the pudendal nerve and enters the perineum through Alcock's canal. The rectal inferior nerve branches off the pudendal nerve in Alcock's canal. The inferior rectal nerve enters the anal canal distally and from lateral through the anal canal wall. (B) We propose that anal sensibility is regulated by nerves that enter the bowel proximally, and from there, they course intramurally from the rectum into the anal canal. The contribution of descending intramural nerves to regulation of anal sensibility: new insights for anorectal surgery. by den Hollander VEC, Br J Surg. 2024 Jan. Copyright 2024 by den Hollander. Reprinted with permission.

The fact that patients born with ARMs could have proper anal sensibility after surgery, whereby the anal canal was completely dissected from the surrounding tissue, indicates that anal sensibility is not regulated exclusively by direct branches of the inferior rectal nerve because this nerve was damaged during surgery. This suggests that a different innervation pattern than currently assumed regulates a substantial part of anal sensibility. This suggested a new innervation pattern, which has recently been demonstrated in a study by the authors (Figure 1) [22]. This newly suggested nerve pathway and the fact that anal sensibility could be present after corrective surgery results in our hypotheses that nerves regulating anal sensibility are embryologically determined and that IAS‐saving procedures result in the preservation of anal sensibility.

Therefore, this study aimed to investigate anal sensibility in our patient cohort in relation to the type of ARM and type of treatment. In addition, we investigated whether anal sensibility was related to the presence of a functional anal canal after corrective surgery. We determined this by examining whether a functional IAS was present after corrective surgery. At last, we investigated whether spinal anomalies were related to anal sensibility.

## 2 Methods

Retrospectively, we searched the database of the Anorectal Physiology Laboratory of the University Medical Center Groningen (APLG), which contains data on patients diagnosed with ARMs who underwent anorectal function tests at our university hospital. All patients that were measured at the APLG were measured due to fecal problems, such as constipation and/or FI. Therefore, this database excludes ARM patients with no fecal problems. We included all patients who, at the time of the measurement, were older than 7 years and thus considered able to perform all function tests [23]. We included patients older than 7 years as the anorectal function tests require the patient to follow instructions and indicate when certain sensations were felt. In addition, all measured patients were cooperative and had no cognitive impairments. Between 2010 and 2022, 68 patients with ARMs underwent anorectal function tests because of constipation and/or FI, and all were included in the study.

We collected information regarding the type of malformation from the medical records according to the Krickenbeck classification [7]. For the analysis, we also categorized patients into two subgroups: minor or major types of ARM. Patients with minor types of ARM were those who were diagnosed with rectoperineal fistula. Patients with major types of ARM were diagnosed with either imperforate anus without fistula, rectourethral bulbar fistula, rectourethral prostatic fistula, recto‐bladder neck fistula, rectovestibular fistula, or cloaca. We collected information on the type of treatment from the patient's medical records, including non‐surgical treatment and the type of surgical intervention. All patients received treatment at our hospital, and those who underwent surgical intervention were all operated at the University Medical Center of Groningen. The patients were treated with the most minimally invasive surgical option. We also collected information about whether patients suffered from abnormalities of the spine and sacrum from the medical records. We calculated the sacral ratio based on AP sacral radiographic images, which are standardly performed after an ARM diagnosis. A sacral ratio of 0.7 is considered normal; a sacral ratio between 0.4 and 0.6 demonstrates a mild abnormality, while a sacral ratio < 0.4 is considered severely abnormal [24, 25, 26]. Furthermore, the Rome IV criteria, collected during follow‐up appointments by the pediatric surgeon, were used to investigate whether patients suffered from constipation. The Medical Ethical Committee of University Medical Center Groningen (METc 2016.054) approved our study. The information collected in this study has been investigated before with another question, which resulted in another article that has been published [22].

### 2.1 Anorectal Function Tests

All patients underwent anorectal function tests as previously described [27]. In all surgically treated patients, the tests were performed after corrective surgery, as these tests can only be performed in cooperative patients, that is, patients who were able to execute the tasks required for the anorectal function tests. All outcomes of the anorectal function tests were re‐evaluated for this study. For this study, three anorectal function tests were performed: the anal mucosal electric sensation test to determine anal sensibility, the RAIR test to determine the existence of the IAS and, therefore, whether the fistula had been transferred or resected during corrective surgery, and the balloon retention test to investigate rectal volumes. Patients received an enema the day before the tests and one at least 2 h before the tests to ensure the anorectum was empty.

### 2.2 Anal Sensibility

Anal sensibility was measured using the anal mucosal electric sensation test. For this test, we used a catheter specifically built for the UMCG and Solar, gastrointestinal, high‐resolution manometry equipment, Version 8.23 (Laborie/Medical Measurement Systems, Enschede, the Netherlands). During this test, a catheter with a diameter of 8F with two circular electrodes 0.8 cm of each other was inserted into the patient's anal canal and connected to a generator that was set to produce a 0.1 ms^2^ wave stimulus at a constant rate of 5 Hz. The current in the electrodes was increased gradually by 1 mA. The current was increased until the patient indicated that the sensation threshold had been reached, usually reported as a burning or tingling sensation. Three threshold measurements were made consecutively, and the lowest repeatable reading was recorded. This procedure was repeated every centimeter starting at 3 cm above the anal verge until just into the anal verge. If no sensation was reported at 20 mA, the test was stopped, and a value of > 20 mA was noted, indicating that the electrosensitivity threshold could not be reached. The higher the values at which the patient reported feeling the electric stimulus, the lower the anal sensibility. The lowest electric stimulus measured at these three levels was chosen for analysis. Electric sensibility of the anal canal between 1 and 7 mA was defined as normal anal sensibility [4]. Electric sensibility between 8 and 19 mA was defined as hyposensibility, and sensibility of 20 mA was considered absent sensibility. The electric stimuli used during this test were so gentle that no muscle contractions were evoked; thus, only anal sensibility, as registered by sensory nerve endings, was measured. Of note, we measured only the electro‐sensibility in the anal canal, and we did not measure rectal sensations because the stimulation thresholds of the rectal wall are much higher than 20 mA.

### 2.3 Internal Anal Sphincter

The RAIR test was used to investigate the presence of a functional IAS. A functional IAS during this test indicated that the fistula had been transferred into the sphincter complex during corrective surgery. A non‐existing recto anal inhibitory reflex means a dysfunctional IAS, and thus demonstrated that the fistula was resected during corrective surgery. During the RAIR test, a catheter with a balloon on top was inserted into the patient's anal canal and rectum. The balloon, positioned in the rectum, was inflated rapidly with air, immediately followed by retracting the air. A decrease of anal rectal pressure of at least 20 mmHg or a decrease of basal anal sphincter pressure of 50% was used to describe the presence of the RAIR and hence the presence of a functional IAS.

### 2.4 Maximum Tolerable Volume

The balloon retention test was used to determine whether patients suffered from a dilated rectum and, thus, constipation. The reason for using this test is that increased rectal volume and constipation are reportedly correlated to reduced anal sensibility [28, 29]. During the balloon retention test, a catheter with a balloon placed on the top of the catheter was inserted into the patient's rectum and gradually filled with 1 mL water of body temperature per second to mimic solid stool. The patients were asked to warn the investigator when they reached the maximum tolerable sensation, after which the test was stopped. The volume at which patients indicated maximum tolerable sensation was used as the maximum volume of the rectum.

### 2.5 Statistical Analysis

We reported values using numbers and means. We analyzed categorical variables using the chi‐squared test. In the case of continuous variables, we used the *t*‐*test* or analysis of variance. Anal sensibility was investigated as a categorical as well as a continuous variable. After testing whether the variables were normally distributed using a Q‐Q plot, we used the Pearson product–moment correlation test to investigate correlation. To examine whether anal sensibility was indeed correlated to the presence of the IAS we performed regression analysis to rule out possible other influential variables. We used linear regression analysis to analyze anal sensibility (dependent variable) with the presence of the RAIR and possible influential variables (independent variables). We postulated that age, sex, type of malformation, sacral ratio, constipation according to the Rome IV criteria, and maximum tolerable volume could affect anal sensibility. A variable with a correlation coefficient with a *p* < 0.1 was selected for multivariable regression analysis or when in previous studies, a variable correlated with anal sensibility. We used stepwise backward elimination to investigate confounders. Variables were eliminated from multivariable analysis when the *p* > 0.05, and the regression coefficient did not change by 10% or more. The overall significance level was preset at a *p* < 0.05.

## 3 Results

### 3.1 Patient Characteristics

Of the 68 ARM patients included in this study, 31 were female (46%). The patients' mean age at the time of the anorectal physiology test was 13 years (SD 3.8, range 7–28). Sixty patients were children, whose mean age was 12 (SD 2.7). The 8 adults had a mean age of 20 (SD 3.4). All the patients were operated in their first year of age. Ten patients were diagnosed with a rectoperineal fistula within the sphincter complex, 15 with a rectoperineal fistula outside the sphincter complex, 4 patients with an imperforate anus without fistula, 9 patients with a rectovestibular fistula, 20 with a rectourethral bulbar fistula, 2 with a rectourethral prostatic fistula, 3 with recto‐bladder neck fistula, and 5 with a cloaca (Table 1). Out of all patients, 7 were treated non‐surgically (all patients were diagnosed with a recto‐perineal fistula), 14 received anterior sagittal anorectal plasty (ASARP), 43 received posterior sagittal anorectal plasty (PSARP), and 4 received other types of treatment, as shown in Table 2.

**TABLE 1:** Anal sensibility per ARM type.

Type of ARM	Number of patients	Mean anal sensibility^a^ in mA (SD)	Number of patients with normal sensibility (1–7 mA)	Number of patients with hyposensibility (8–19 mA)	Number of patients with absent sensibility (> 19 mA)
Total	68	7.2 (5.2)	48	15	5
Minor	25	5.1 (4.3)*	21	3	1
Major	43	8.4 (5.4)*	27	12	4
Imperforate anus without fistula	4	9.3 (3.8)	1	3	0
Recto‐perineal fistula within the sphincter	10	3.1 (1.3)	10	0	0
Recto‐perineal fistula outside the sphincter	15	6.5 (5.1)	11	3	1
Recto‐vestibular fistula	9	5.8 (2.2)	8	1	0
Recto‐urethral bulbar fistula	20	9.9 (5.7)	10	8	2
Recto‐urethral prostatic fistula	2	12.0 (11.3)	1	0	1
Recto‐bladder neck fistula	3	5.0 (1.0)	3	0	0
Cloaca	5	7.6 (7.1)	4	0	1

^a^ The lowest electric stimulus was used to calculate anal sensibility.

* Significantly different, *p* = 0.007.

**TABLE 2:** Anal sensibility according to the type of treatment.

Type of surgery	Number of patients	Mean anal sensibility in mA (SD)	Normal sensibility (1–7 mA)	Hypo sensibility (8–19 mA)	Absent sensibility (> 19 mA)
Type of ARM					
Non‐surgical	7	2.7 (1.3)	7	0	0
Recto perineal fistula	7				
Bucket handle	1	3.0	1	0	0
Recto perineal fistula	1				
Cutback	1	9.0	0	1	0
Recto perineal fistula	1				
Anoplasty	1	3.0	1	0	0
Recto perineal fistula	1				
ASARP	14	6.1 (5.3)	11	2	1
Recto perineal fistula	10				
Recto vestibular fistula	4				
PSARP	39	8.5 (5.3)	27	12	4
No perineal fistula	4				
Recto perineal fistula	5				
Recto vestibular fistula	5				
Recto urethral bulbar fistula	20				
Recto urethral prostatic fistula	2				
Recto‐bladder neck fistula	2				
Cloaca	5				
LAARP	1	5.0	1	0	0
Recto‐bladder neck fistula	1				
Total	68	7.2 (5.2)	48	15	5

#### 3.1.1 Anal Sensibility in Relation to the Type of Anorectal Malformation

**FIGURE 2:**
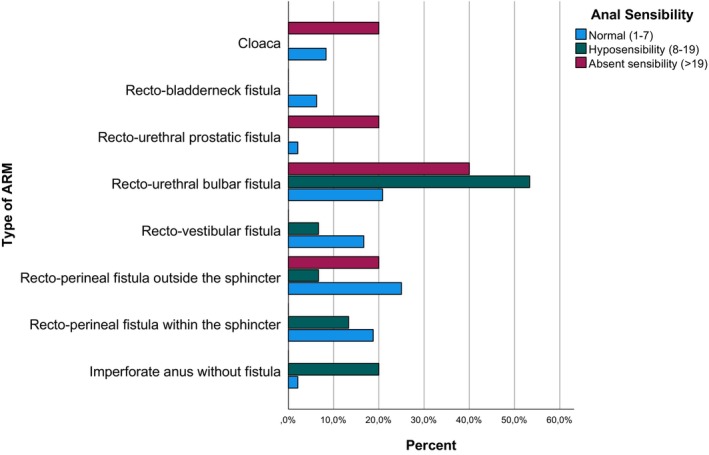
Anal sensibility per ARM type.

Overall, the mean anal sensibility was 7.2 mA (SD 5.2). Out of all patients, 48 were categorized as having normal anal sensibility, 15 suffered from hyposensibility, and 5 lacked anal sensibility. Normal recordings of anal sensibility were observed in patients with all types of ARM, even in patients diagnosed with more severe types of ARM. Furthermore, we found that patients with a minor ARM had a significantly lower anal electrosensitivity threshold than patients with a major ARM (5.1 vs. 8.4 mA, respectively, *p* = 0.007) (Table 1). Anal sensibility parameters stratified for the specific type of ARM is presented in Table 1, Figure 2.

#### 3.1.2 Anal Sensibility in Relation to the Type of Treatment

The patients who had either been treated non‐surgically, who had undergone a bucket handle procedure, an anoplasty, or a laparoscopic‐assisted anorectoplasty had normal anal sensibility. Among the patients who had undergone ASARP, 3 out of 14 patients had impaired anal sensibility, 2 of whom suffered from hyposensibility and 1 lacked anal sensibility. Out of the 43 patients treated with PSARP, 12 were diagnosed with hyposensibility, and in 4 patients, anal sensibility was absent. Those treated non‐surgically had significantly better anal sensibility than those treated surgically (2.7 vs. 7.7 mA, respectively, *p* = < 0.001). The outcomes are presented in Table 2.

#### 3.1.3 Anal Sensibility in Relation to Anorectal Physiology in the Distal End of the Bowel

Of all the patients, 48 (71%) had a RAIR. Interestingly, patients who possessed a RAIR, sensed significantly lower electric stimuli than patients who did not possess RAIR, 6.4 and 9.2 mA, respectively (*p* = 0.038) (Table 3). Anal sensibility correlated significantly with the presence of a RAIR (*R* = −0.25, *p* = 0.038), the presence of constipation (*R* = −0.36, *p* = 0.003), sacral ratio (*R* = −0.25, *p* = 0.056), sex (*R* = −0.22, *p* = 0.07) and type of ARM (*R* = −0.21, *p* = 0.09). Anal sensibility did not correlate with age (*R* = −0.15, *p* = 0.23) and maximum tolerable volume (*R* = −0.16, *p* = 0.19). Multivariable linear regression showed associations between anal sensibility and the presence of a RAIR (st *B* = −0.22, *p* = 0.06) and the presence of constipation (st *B* = 0.34, *p* = 0.004) after we statistically corrected for sex, type of malformation, sacral ratio, and maximal tolerable volume.

**TABLE 3:** Anal sensibility according to the rectoanal inhibitory reflex.

Presence of RAIR^a^	Number of patients	Mean anal sensibility	*p*
RAIR+	48	6.38 (SD 5.127)	0.038
RAIR−	20	9.25 (SD 5.014)	

a Rectoanal inhibitory reflex.

#### 3.1.4 Anal Sensibility in Relation to the Sacral Ratio

The sacral ratios of 60 patients were available. Of the 60 patients, 6 suffered from a sacral ratio < 0.4, 14 had a sacral ratio between 0.4 and 0.6, and 40 had a sacral ratio > 0.6. The outcomes are demonstrated in Table 4. Anal sensibility seems to be better in those with a sacral ratio > 0.6. However, this outcome was not significantly different. In addition, patients with all types of ARM could have a normal sacral ratio (> 0.6). A sub‐analysis of the patients with a sacral ratio < 0.4 demonstrated that of the six patients, four were diagnosed with a recto‐urethral fistula, one with a cloaca, and one with a recto‐perineal fistula. Of these six patients, five had a functional IAS, and three patients had normal anal sensibility, two had diminished anal sensibility, and one had no anal sensibility (Table 5).

**TABLE 4:** Sacral ratio in relation to anal sensibility.

Sacral ratio	*N*	Type of ARM (*n*)	Anal sensibility category (*n*)	Mean anal sensibility	*p*
< 0.4	6	Recto‐perineal fistula outside the sphincter (1) Recto‐urethral bulbar fistula (4) Cloaca (1)	Normal sensibility (3) Hyposensibility (2) Absent sensibility (1)	10.0 (6.5)	0.24
0.4–0.6	14	Recto‐perineal fistula inside the sphincter (1) Recto‐perineal fistula outside the sphincter (2) Recto‐vestibular fistula (4) Recto‐urethral bulbar fistula (4) Recto‐urethral prostatic fistula (1) Recto‐bladder neck fistula (1) Cloaca (1)	Normal sensibility (9) Hyposensibility (3) Absent sensibility (2)	8.2 (5.7)	
> 0.6	40	Imperforate anus without fistula (2) Recto‐perineal fistula within the sphincter (8) Recto‐perineal fistula outside the sphincter (9) Recto‐vestibular fistula (4) Recto‐urethral bulbar fistula (11) Recto‐urethral prostatic fistula (1) Recto‐bladder neck fistula (2) Cloaca (3)	Normal sensibility (32) Hyposensibility (6) Absent sensibility (2)	6.4 (5.1)	

**TABLE 5:** Patients with a sacral ratio < 0.4.

ID	Type of ARM	Anal sensibility	RAIR
48	Recto‐urethral	20	+
27	Recto‐urethral	14	−
19	Recto‐perineal	4	+
2	Cloaca	6	+
21	Recto‐urethral	4	+
58	Recto‐urethral	12	+

## 4 Discussion and Conclusions

Anal sensibility is an essential mechanism for maintaining fecal continence but has been studied scarcely in patients diagnosed with ARM. Only one study by Ikeda et al. investigated anal sensibility in different types of ARM [2]. They demonstrated that anal sensibility could be present in low, middle, and high types of ARM. They showed that patients diagnosed with low types of ARM had better anal sensibility than those diagnosed with middle and high types of ARM. This is in comparison with the outcomes demonstrated in this study.

Furthermore, we also found that patients who were treated non‐surgically or underwent less extensive corrective surgery had better anal sensibility than those treated with extensive surgery. These outcomes suggest that those diagnosed with minor types seem to have better anal sensibility than those with major types. However, those diagnosed with minor types underwent less extensive types of surgery. As anal sensibility was present in all types of ARM and the fact that those who had been treated non‐surgically had good anal sensibility suggests that nerves regulating anal sensibility are embryologically determined in patients born with ARM. Thus, it seems that diminished anal sensibility is the result of corrective surgery. However, the exact nerve pathway has only recently been described [22].

To date, the exact innervation pattern and mechanisms that regulate anal sensibility have not been confirmed. Various studies believed that the inferior rectal nerve regulated anal sensibility. In 1960, Duthie and Gairns were the first to demonstrate that the anal canal contains free and organized nerve endings. In 1976, Gunterberg et al. showed that the loss of bilateral nerves rooting from S2 and S3 resulted in the loss of anal sensibility [30, 31]. Hereafter, four studies investigated the anatomy of the pelvic floor and the various nerve pathways of the anorectum [6, 19, 32, 33]. They investigated the pudendal and inferior rectal nerves and believed that these nerves are responsible for the sensory part of the anal canal. However, we demonstrated in our earlier study that a part of anal sensibility must be regulated by nerves traveling from the proximal bowel transmurally into the anal canal (Figure 1) [22]. We found that patients born with an ARM can have normal anal sensibility even though their fistula, that is, the ectopic anal canal, had been completely dissected from the surrounding tissue.

As demonstrated in Figure 1, it is, therefore, not possible that the inferior rectal nerves alone account for anal sensibility because the inferior rectal nerves that enter the anal canal distally from the lateral direction incurred damage during surgery (Figure 1A). This proposition is supported by the fact that we, in the present study, demonstrate that the RAIR, which is regulated by intramural nerves that innervates the IAS, is moderately associated with anal sensibility. Patients who did not possess a functional IAS after corrective surgery, that is, patients who had undergone surgery whereby the fistula was resected, had a higher electrosensitivity threshold than those who had undergone surgery whereby the fistula, including the IAS, was saved and placed within the external sphincter complex. Thus, it seems that the fistula contains not only a part of the IAS but also sensory nerves that regulate anal sensibility (Figure 1B). In this way, we support the conclusions of previous authors who stated that the fistulous ending of the bowel is, in fact, an ectopic anal canal [14, 15]. Consequently, we present additional evidence that this section of the ectopic anal canal should not be resected. Although the nerve pathway that is proposed seems to be the only possible route for nerves that regulate anal sensibility, one might assume that it is also possible that after damage to the inferior rectal nerves, the nerves grow back through the anal canal wall and that in this way it regulates anal sensibility at an older age. This proposition is unlikely because we cannot explain why these nerves would only grow back into the anal canal of patients who possess an IAS and why they would not grow back in patients who do not have an IAS. Altogether, we advise preserving the “fistula” during surgical procedures as much as possible. From clinical experience of our clinic, we have no evidence that preservation of this section has adverse outcomes, such as anal stenosis, for example.

We confirmed that anal sensibility did not correlate with sex and age, corroborating findings from other studies [28, 34]. We also demonstrated that sacral ratio was not significantly related to anal sensibility even though the outcomes suggest that patients with a normal sacral ratio have better anal sensibility. Furthermore, the sub‐analysis demonstrated that those with a sacral ratio < 0.4 were almost all diagnosed with a recto‐urethral fistula, and 50% had diminished anal sensibility. Thus, the sacral ratio might be related to the type of ARM and diminished anal sensibility. On the other hand, the fact that those with a sacral < 0.4 could have normal anal sensibility demonstrates that an abnormal sacrum and/or spine does not seem to interfere with the neural pathway that regulates anal sensibility. However, it is also possible that the nerves regulating anal sensibility derive from above the sacrum. In this way, an anomaly of the sacrum would not affect anal sensibility. Also, our cohort's relatively small sample size might have contributed to this outcome. As we only included six patients with a sacral ratio < 0.4 we cannot make hard conclusions. Furthermore, we found that patients with a recto‐urethral fistula could have a sacral ratio > 0.6 and could have normal anal sensibility. Also, those diagnosed with major types of ARM (recto‐bladder neck and cloaca) could have normal sacral ratios and normal anal sensibility. Statistical analysis using both the chi‐square test and multivariable regression analysis demonstrated no significant relation. Therefore, the outcomes of this study seem to prove that sacral ratio is not directly related to the type of ARM and/or anal sensibility.

We found that constipation was associated with decreased anal sensibility. The results of this study showed that decreased anal sensibility was also related to the absence of the IAS. In one of our previous studies, we demonstrated that the absence of the IAS is correlated with constipation in patients with ARM. Since the internal sphincter and anal sensibility seem to be located in the fistula and both will be resected during the operation, it explains why constipation is related to decreased anal sensibility [13].

### 4.1 Limitations

Even though the study improved our knowledge of the physiology of the anorectum of ARM patients, the study has several limitations. First, because ARM belongs to rare diseases with a prevalence of 1:5000, it was not feasible to include a more extensive study population, which might have biased our statistical outcomes. Due to this study's retrospective character, some data were also missing. Moreover, for this retrospective study, we assessed medical records of patients who had been referred to the Anorectal Physiology Laboratory due to existing anorectal problems and, consequently, were able to include patients who suffered from fecal issues such as constipation and FI and fewer patients without these anorectal problems. Hence, this study would benefit from including patients without fecal problems. It would strengthen our assumption that patients would benefit from corrective surgery that keeps as much of the distal end of the bowel (the fistula) intact as possible while reconstructing a neo‐anus.

### 4.2 Clinical Implications

The outcomes of this study will have several consequences for patients with ARM and all patients who need surgery in the anorectal region. First of all, this study demonstrates that in the case of ARM patients, the distal end of the fistula should be spared as much as possible to protect against damage to the IAS and anal sensibility. Practically, this means that the distal end of the bowel should be transferred as entirely as possible into the external anal sphincter during corrective surgery. Second, if patients with ARM suffer from FI after corrective surgery, anorectal function tests should point out whether the patient is suffering from diminished anal sensibility. In such cases, complete fecal continence will be rigid to establish, but treatment options are available, such as pelvic physical therapy and/or a bowel management program.

Lastly, these newly discovered outcomes will also have consequences for all patients who need surgery in this area. For example, patients that need to undergo low anterior resection, may benefit from surgery that spares the nerves that regulate anal sensibility. A study of Verkuijl et al. demonstrated that patients who underwent low anterior resection suffered from FI when the resection was within 5 cm of the anal canal [35]. Those that had a resection further from the anal canal suffered no FI. Even though Verkuijl et al. did not investigate anal sensibility, this outcome may suggest that the nerves regulating anal sensibility got damaged during the resection when the resection was within 5 cm of the anal canal. Therefore, it is of the utmost importance to investigate the exact nerve pathway further so that all patients who receive anorectal surgery will not suffer FI.

In conclusion, this study proves that anal sensibility can be present in patients born with ARM, irrespective of the malformation type. In addition, we prove that the distal end of the bowel in ARM patients, the fistula, is, in fact, an ectopic anal canal that contains both the IAS and the nerves that regulate anal sensibility. Therefore, it is of the utmost importance to keep as much of the distal end of the bowel (the fistula) intact as possible while reconstructing a neo‐anus in these patients. In this way, even patients diagnosed with the most severe types of ARM may improve over time and show good fecal continence.

Overall, the outcomes of this study enhance our understanding of the physiology of the anorectum of ARM patients and the embryology of ARM, which may result in a new surgical strategy. Moreover, our findings have broader applicability, as they contribute to the existing understanding of the relationship between the IAS and anal sensibility. This knowledge will impact all procedures performed in the anorectal region. We recommend further research, including a neuroanatomical study to elucidate the precise neural pathways underlying the association between the IAS and anal sensibility. Additionally, a prospective study is required to determine whether patients with preserved anal sensibility experience better long‐term outcomes in terms of fecal continence and defecation. Such studies, and the subsequent application of our findings in clinical practice, require collaboration between surgeons, gastroenterologists and researchers, as well as open dialogue between specialists from these disciplines.

## Author Contributions

Venla E.C. den Hollander conducted the study, interpreted and analyzed the data, and drafted, reviewed and revised the manuscript. Monika Trzpis conducted the study, interpreted the data, and critically reviewed the manuscript for important intellectual content. Paul M.A. Broens designed the study, collected and interpreted the data, and critically reviewed the manuscript for important intellectual content. All authors approve the final manuscript and agree to be accountable for all aspects of the work.

## Conflicts of Interest

The authors declare no conflicts of interest.

## Data Availability

Data can be made available upon reasonable request. Requests can be sent to p.m.a.broens@umcg.nl.
